# Using Android and Open Data Kit Technology in Data Management for Research in Resource-Limited Settings in the Niger Delta Region of Nigeria: Cross-Sectional Household Survey

**DOI:** 10.2196/mhealth.7827

**Published:** 2017-11-30

**Authors:** Omosivie Maduka, Godwin Akpan, Sylvester Maleghemi

**Affiliations:** ^1^ Department of Preventive and Social Medicine College of Health Sciences University of Port Harcourt Port Harcourt Nigeria; ^2^ World Health Organization Port Harcourt Nigeria

**Keywords:** mobile phones, technology, Africa

## Abstract

**Background:**

Data collection in Sub-Saharan Africa has traditionally been paper-based. However, the popularization of Android mobile devices and data capture software has brought paperless data management within reach. We used Open Data Kit (ODK) technology on Android mobile devices during a household survey in the Niger Delta region of Nigeria.

**Objective:**

The aim of this study was to describe the pros and cons of deploying ODK for data management.

**Methods:**

A descriptive cross-sectional household survey was carried out by 6 data collectors between April and May 2016. Data were obtained from 1706 persons in 601 households across 6 communities in 3 states in the Niger Delta. The use of Android mobile devices and ODK technology involved form building, testing, collection, aggregation, and download for data analysis. The median duration for data collection per household and per individual was 25.7 and 9.3 min, respectively.

**Results:**

Data entries per device ranged from 33 (33/1706, 1.93%) to 482 (482/1706, 28.25%) individuals between 9 (9/601, 1.5%) and 122 (122/601, 20.3%) households. The most entries (470) were made by data collector 5. Only 2 respondents had data entry errors (2/1706, 0.12%). However, 73 (73/601, 12.1%) households had inaccurate date and time entries for when data collection started and ended. The cost of deploying ODK was estimated at US $206.7 in comparison with the estimated cost of US $466.7 for paper-based data management.

**Conclusions:**

We found the use of mobile data capture technology to be efficient and cost-effective. As Internet services improve in Africa, we advocate their use as effective tools for health information management.

## Introduction

### Rationale

Data collection may be of a routine nature such as from registers or ad hoc such as from evaluations or research activities. Effective data management is essential for evidence-based public health interventions and contributes to sustainable development. For a data collection effort to meet the needs of policy makers, projects, programs, and other stakeholders, it must be comprehensive, accurate, cost-effective, and timely.

Data collection for research purposes in many locations in Sub-Saharan Africa has been traditionally paper-based [1]. Questions are typed on data processing machines and multiple copies printed and photocopied based on the sample size. These are self-administered or interviewer-administered, and information filled in these data sheets/questionnaires are manually entered into data processing software and analyzed. This strategy is fraught with several shortcomings spanning all stages of data management. During data collection, issues of nonresponse to sections of the tool, data entry errors, and false data entries are possibilities. Data collation may also be subject to errors during transfer to databases. These necessitate that a great deal of person-hours are spent in *data cleaning*, which is the painstaking process of correcting or removing incorrect, duplicate, or corrupt data entries in an attempt to improve the validity of research findings. Data collation and cleaning is especially challenging when dealing with large sample sizes [2].

Mobile-based data capture software attempt to resolve these problems and improve the validity and reliability of health information. Data capture software are linked to databases, servers, or repositories for easy uploads and retrieval to the data analysis software, thus eliminating the need for data entry. Furthermore, many of them also have inbuilt data quality checks that improve data completeness and accuracy. Although various versions of the software have been in existence for decades and are actively used by researchers in developed countries, its usefulness has only recently been explored in resource-limited settings [1,3-10].

The growing popularity of Android mobile devices in Sub-Saharan Africa, the explosion in the telecommunications market, and the development of user-friendly mobile apps have brought mHealth within reach of populations in Sub-Saharan Africa. Nigeria, with a population of over 187 million people, has been a haven for the expansion of telecommunication and mobile phone companies. These companies have flooded the market with affordable brands of Android mobile phones with the cheapest going for as low as US $30.00. Furthermore, these devices can be used multiple times over many years, making it a cost-effective tool for data collection as part of research efforts.

In spite of this, use of mobile technology for research is not very popular among researchers in the country. This is because its use is perceived to be cost-intensive, heavily reliant on information technology expertise, and cumbered by unstable electricity and Internet connectivity. As such, it is often restricted to use by international nongovernmental agencies. These perceived bottlenecks can, however, be quickly overcome so that researchers are able to enjoy their obvious benefits.

### Objective

As part of research efforts during the Department for International Development (DfID)–funded Climate Impact Research, Capacity and Leadership Enhancement (CIRCLE) research fellowship, we undertook to use Open Data Kit (ODK) technology. ODK was first developed by researchers at the University of Washington’s Department of Computer Science and Engineering in 2008. It has enjoyed a wide application of uses in epidemiology. We implemented its use for data management in a community-based household survey to assess and compare morbidity and mortality patterns between persons residing in communities exposed to gas-flaring and those residing in communities without any oil exploration activities. We sought to characterize the pros and cons of using the ODK software and Android technology for data management in a large study, in terms of personnel, accuracy, time, and cost.

## Methods

### Study Design and Study Area

A descriptive cross-sectional household community survey was carried out over a three-week period between April and May 2016 among a sample of 1706 persons spanning 600 households in 6 communities in 3 states in the Niger Delta region of Nigeria. The Niger Delta region of Nigeria, as defined by the Nigerian Government, occupies about 70,000 km² and makes up 7.5% of Nigeria’s landmass. It comprises the 9 oil-producing states in the country, namely, Bayelsa, Rivers, Delta, Akwa Ibom, Cross River, Edo, Abia, Imo, and Ondo. These states are home to about 31 million people spanning over 40 ethnic groups and 185 Local Government Areas (LGAs), who speak about 250 different dialects. The study sites were in Mbodo-Aluu and Omuhiombia in Ikwerre LGA of Rivers State, Ibada-Elume and Oton in Sapele LGA of Delta State, Sampou in Kolokuma/Opokuma LGA, and Nedugo in Yenagoa LGA in Bayelsa State.

### Study Instrument and Data Collection

**Figure 1:**
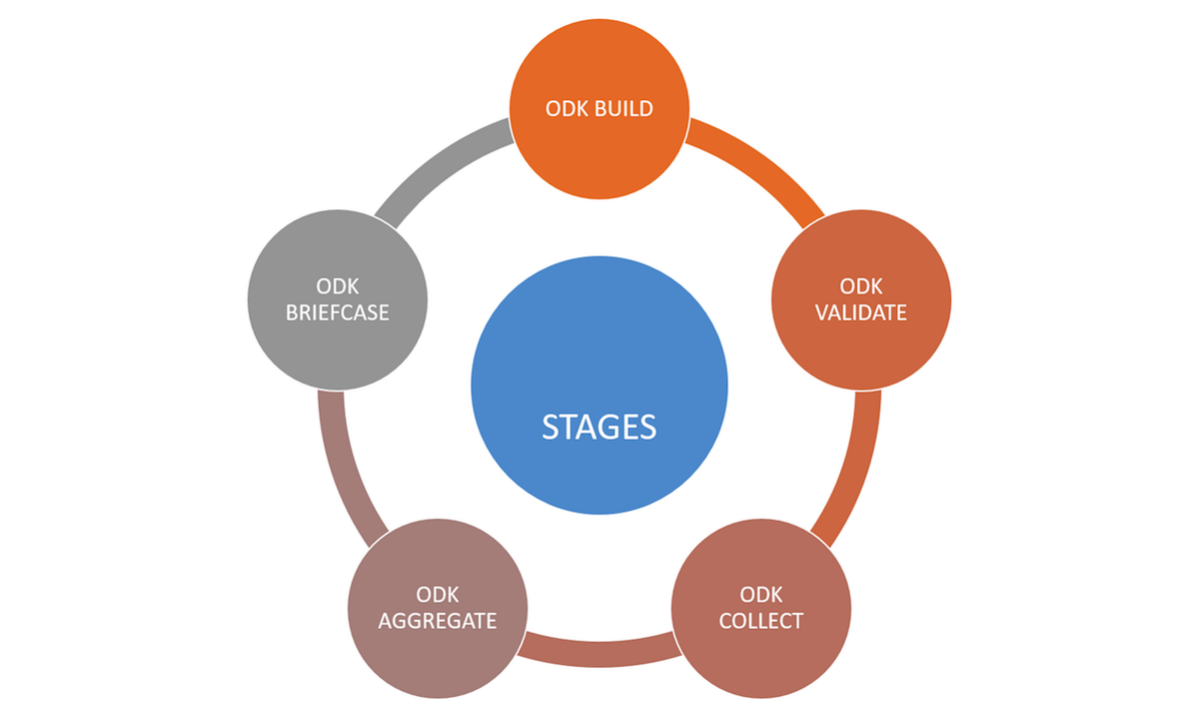
Stages in deployment of Open Data Kit for data collection.

The use of ODK uploaded on Android mobile devices involved the following steps (Figure 1): form building, validation/testing, training, data collection, collation (aggregate and briefcase), and data analysis. The original household questionnaire was a 15-page, 145-item interviewer-administered tool in 7 subsections. Building of this form on ODK included a consent page for collecting signatures, indicating that informed consent has been given; global positioning system (GPS) capturing of household coordinates; sociodemographic information; biological measurements; and information on household environmental characteristics, morbidity, and mortality.

Six field data collectors participated in a 1-day training on the use of the e-questionnaires that had been uploaded onto the ODK collect application downloaded onto Android mobile phones. All the data collectors were experienced in the use of Android mobile phones. However, only 1 data collector was experienced with using the ODK collect application on Android phones. The training covered familiarization with ODK collect application, the e-questionnaire, how to take GPS coordinates, administration of informed consent, and taking biological measurements. The researchers facilitated various aspects of the 1-day training.

Eight Android mobile phones were granted by the World Health Organization (WHO) state office for data collection. The extra 2 phones served as backup in the event of malfunction or challenges with global system for mobile communication (GSM) mobile and data networks.

Each data collector was assigned a target of 10 households per day per community. Field data collectors were trained to upload completed forms onto a secure server, with back-end access provided to only the research team lead. Data underwent “cleaning” to identify errors. Inaccurate date and time records were identified during data cleaning through a review of the start/end date and time records from the device. The data entries underwent a preliminary analysis on the server platform before downloading in Microsoft Excel format and exporting to SPSS version 21 (IBM Corp) for analysis.

Ethical approval for the research was obtained from the research ethics committee of the University of Port Harcourt (ethics approval number: UPH/CEREMAD/REC/04). In addition, each community gave permission for data collection through the community leaders.

## Results

### Characteristics of ODK Data Collection

A total of 8 ODK-enabled Android mobile phones were used by 6 data collectors to collect data from 1706 household members in 601 households. Data entries per device ranged from 33 (1.93%, 33/1706) to 482 (28.25%, 482/1706) among individuals and between 9 (1.5%, 9/601) and 122 (20.3%, 122/601) households. The most entries (470) were made by data collector 5 using only 1 device (Tables 1 and 2). In addition, there were only 2 respondents (0.12%, 2/1706) with missing data out of the 1706 persons recruited into the study.

**Figure 2:**
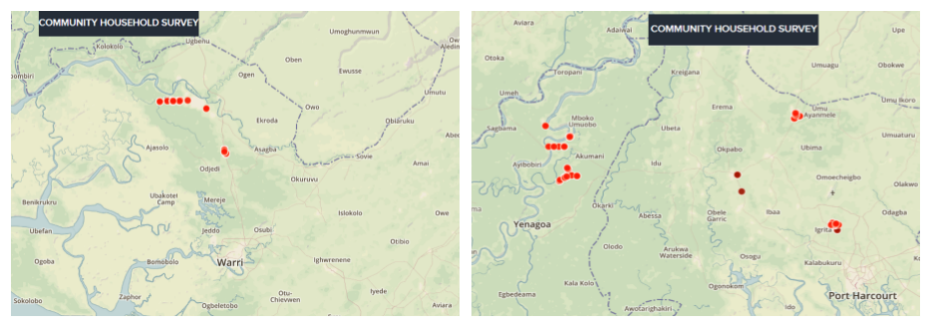
Geomap of all the households across the 6 communities in 3 states of the Niger Delta region of Nigeria.

ODK phones obtained GPS coordinates for all 601 households in the study communities. Using this, we were able to plot the geomap illustrating the location of each household across the study communities (Figure 2).

**Table 1:** Number of data entries for households and household occupants per device (N=1706).

Device ID	Households (N=601), n (%)	Individuals (N=1706), n (%)
3523xxxxxxxxxxxx	107 (17.8)	303 (17.76)
3538xxxxxxxxxxxx	120 (20.0)	168 (9.85)
3538xxxxxxxxxxxx	122 (20.3)	482 (28.3)
3538xxxxxxxxxxxx	109 (18.1)	303 (17.76)
3538xxxxxxxxxxxx	10 (1.7)	34 (1.99)
3560xxxxxxxxxxxx	26 (4.3	76 (4.45)
8628xxxxxxxxxxxx	9 (1.5)	33 (1.93)
8636xxxxxxxxxxxx	98 (16.3)	307 (18.00)

**Table 2:** Devices used by data collectors, number of households, and data entries made.

Interviewer and devices used		Number of entries (N=1705)	Number of households (N=601)
**Interviewer 1**		**N=310**	**N=110**
	3523xxxxxxxxxxx	303	107
	3560xxxxxxxxxxx	5	2
	8636xxxxxxxxxxx	2	1
**Interviewer 2 **		**N=46**	**N=13**
	3538xxxxxxxxxxx	2	1
	3538xxxxxxxxxxx	9	2
	3560xxxxxxxxxxx	2	1
	8628xxxxxxxxxxx	33	9
**Interviewer 3**		**N=166**	**N=119**
	3538xxxxxxxxxxx	166	119
**Interviewer 4**		**N=340**	**N=120**
	3538xxxxxxxxxxx	3	1
	3538xxxxxxxxxxx	303	109
	3538xxxxxxxxxxx	34	10
**Interviewer 5**		**N=470**	**N=119**
	3538xxxxxxxxxxx	470	119
**Interviewer 6**		**N=374**	**N=120**
	3560xxxxxxxxxxx	69	23
	8636xxxxxxxxxxx	305	97

**Table 3:** Accuracy of date and time for Android mobile phones used for data collection (N=number of households where data were collected).

Device identity	Frequency (N=601)	
	Accurate timing (N=528), n (%)	Errors in date/time (N=73), n (%)
3523xxxxxxxxxxx (N=107)	106 (99.1)	1 (0.9)
3538xxxxxxxxxxx (N=120)	110 (91.7)	10 (8.3)
3538xxxxxxxxxxx (N=122)	102 (83.6)	20 (16.4)
3538xxxxxxxxxxx (N=109)	83 (76.1)	26 (23.9)
3538xxxxxxxxxxx (N=10)	6 (60)	4 (40)
3560xxxxxxxxxxx (N=26)	24 (92)	2 (8)
8628xxxxxxxxxxx (N=9)	9 (100)	0 (0)
8636xxxxxxxxxxx (N=98)	88 (90)	10 (10)

### Date and Time Characteristics

Of the data collected using 8 devices over 601 households, 528 households (87.9%) had accurate date and time recordings for when data collection started and ended, whereas 73 (12.1%) had errors in date or time of data collection (Table 3). Of these, 20 (27.5%) errors were from an error in date settings on 1 mobile device, 2 (2.7%) errors were from an absence in start time, and the remaining 51 (69.8%) errors resulted from inability to obtain GPS coordinates (due to network down time) on one day, necessitating repeat visits the next day. Those forms could not be completed until all the required fields had been filled.

Only households with accurate date and time records were used (n=528) in calculating mean and median duration of data collection. The average duration of data collection for all households analyzed was 55.8 (standard deviation, SD=73.3) min with a median duration of 25.7 min. The shortest average duration of data collection was at Omerelu (25 min), whereas the longest average duration for data collection was at Oton (89.5 min). An average of 29.2 (SD 9.3) min was used for individual interviews with a median duration of 9.3 min (Table 4).

**Table 4:** Summary statistics for time spent collecting data using Open Data Kit across households and individuals in each community and for each Android mobile device.

Characteristics			Households						Individuals				
			Duration, mean (SD)		Median		Range		Duration, mean (SD)		Median		Range
**Communities**													
	Ibada-Elume (n=99)	41.1 (56.8)		21.1		6.9-395.7		23.8 (35.5)		8.0		3.0-161.9	
	Mbodo (n=73)	74.9 (94.2)		37.8		11.1-416.7		34.7 (56.8)		14.0		4.3-400.6	
	Nedugo (n=94)	48.9 (55.9)		25.5		8.5-263.4		26.1 (41.9)		8.0		3.2-202.5	
	Omerelu (n=85)	25.0 (9.9)		22.7		8.7-57.7		10.2 (5.7)		8.0		3.0-25.7	
	Oton (n=78)	9.5 (105.6)		30.7		8.1-374.3		50.7 (74.4)		13.3		3.1-374.3	
	Sampou (n=99)	62.9 (70.9)		28.8		8.9-322.7		33.1 (45.6)		9.2		2.4-193.5	
**Android devices**													
	3523xxxxxxxxxxx (n=106)	53.2 (31.2)		19.9		6.9-416.7		21.2 (9.2)		9.2		3.2-208.4	
	3538xxxxxxxxxxx (n=110)	100.2 (90.8)		73.5		8.3-400.6		79.1 (74.8)		57.7		8.3-400.6	
	3538xxxxxxxxxxx (n=102)	41.7 (40.6)		28.8		14.0-271.6		13.4 (19.7)		7.2		3.2-135.8	
	3538xxxxxxxxxxx (n=83)	37.9 (39.6)		23.3		10.7-217.1		16.8 (24.6)		8.4		3.9- 158.8	
	3538xxxxxxxxxxx (n=6)	29.3 (9.8)		31.8		14.6-38.7		7.9 (1.9)		8.3		4.9-9.7	
	3560xxxxxxxxxxx (n=24)	17.2 (8.6)		14.2		8.9-47.0		6.4 (3.2)		5.6		2.4-16.40	
	8628xxxxxxxxxxx (n=9)	194.4 (104.0)		181.6		46.6-322.7		55.7 (30.0)		60.5		11.6-91.0	
	8636xxxxxxxxxxx (n=88)	34.8 (58.4)		18.8		8.1-368.0		11.7 (18.4)		6.6		3.0-122.7	
All data collection (n=528)			55.8 (73.3)		25.7		6.9-416.7		29.2 (9.3)		9.3		2.4-400.6

**Table 5:** Implementation cost (comparing cost elements between paper-based and Open Data Kit phone).

Stages of data management	Android-based mobile devices (US $)	Paper-based (US $)	Comments
Questionnaire design and pretesting	0	0.0	This was done by the investigators
Purchase/hire of Android mobile phones	0 (300)^c^	0.0	Mobile phones were granted for data collection by the WHO^a^ state program office
Building of forms on ODK^b^ platform	66.7	0.0	
Training of data collectors	33.3	33.3	
Mass production of questionnaires of 15 pages (2000 copies)	0.0	100.0	US $0.03 per page for 15 pages and 2000 copies
Data entry and cleaning	66.7	333.3	Data cleaning alone needed for ODK^b^
Data download and cleaning	0.0	0.0	
Airtime and Internet data plans	40.0	0.0	US $5.0 per device
Total estimated costs	206.7 (506.7)^c^	466.7	

^a^WHO: World Health Organization.

^b^ODK: Open Data Kit.

^c^Cost of deployment, should the project have needed to purchase the same quality of Android phones obtained for use from the WHO state office.

### Comparing Cost of ODK and Paper-Based Data Collection

A cost analysis conducted showed the costs of deploying ODK on Android mobile phones for data management to be US $206.7 compared with the cost of paper-based data management of US $466.7 using already existing mobile phones. However, when the cost analysis factored in phone purchases, the cost of deploying ODK increased to US $506.7. The major contributors to the cost of using ODK on Android mobile phones, aside from purchase of the phone, included building of the forms, back-end data management, and Internet access. The major cost considerations for paper-based data management are data entry and cleaning and mass production of the data sheets (Table 5).

## Discussion

### Principal Findings

Our study identified some pros and cons for the use of ODK software on Android mobile devices for data collection in a resource-limited setting. The pros included a comparatively shorter time on the field, improved rigor and accuracy of data collection, effective collection of GPS data and consent signatures, real-time monitoring of data entries, and reduced time lag between data collection and analysis in comparison with paper-based data collection. The cons we identified were related to the quality of the Android phones, errors in date and time recorded for interviews on some Androids, challenges with fluctuations in the GSM mobile and data networks leading to some delays in completing and uploading forms, and the increase in implementation costs if we were to purchase Android phones rather than leverage on existing ones. Our ability to leverage on existing/available Android devices facilitated implementation costs that were lower than what would have been incurred on paper-based data management. We also took advantage of scarce expertise in programming for building the forms and providing technical assistance in the use of the ODK collect and Ona platforms. Another downside of using ODK on Android mobile phones from our experience was that some interviews seemed to take as much as 7 hours to complete. This resulted from the fact that a few data collectors encountered challenges with Internet connectivity necessary for capturing GPS coordinates.

Android-based mobile phones offer additional capabilities, including built-in GPS functionality and other applications that can be integrated into electronic data collection such as digital photography and automated timestamp information. Mobile information technology also enables other data sources, including census and mapping data, and other tools for data visualization, including Google Maps, to be more readily integrated into the process of data collection, reporting, and analysis [3,11,12]. The multiple uses of mobile devices for message and calls, social media interactions, and data capture and management among others makes it a very useful tool for virtually all public health interventions. However, the quality of the Android mobile device comes into play as a factor affecting accuracy of data collection especially with regard to accuracy of GPS coordinates and data and time settings.

### Limitations

The challenge related to the cost of the Android mobile device can be surmounted by uploading the ODK software on the Android devices owned by the research team and data collectors, thus eliminating the need to purchase Android phones. Researchers will, however, need to be deliberate about using medium-to-high grade Android phones so as to eliminate errors such as those we experienced with date and time on some of the phones used. Cellular and data service providers often do not have coverage in certain communities, and this can provide a limitation to data collection with Android phones. Fluctuations in Internet service may also lead to delays in completing data entry and uploading completed forms. This limitation played out in our study such that some Android devices recorded as much as 7 hours of interview for some households because of poor data connection to finalize and upload data forms. We attempted to manage this limitation by providing multiple subscriber identity module (SIM) cards for each data collector depending on the cellular networks available in the community. However, in some situations, we resorted to repeat visits to the households to collect GPS coordinates, whereas in others, we collected the data offline and uploaded it later once data network was restored [5].

There were differences between the number of interviews conducted between devices and data collectors. This can be accounted for by the use of 8 mobile phones by 6 data collectors in the course of data collection. The extra phones came in handy when a phone malfunctioned or when there were network outages affecting one service provider and necessitating a switch to another service provider.

ODK software is one of the several mobile-based data management software and e-platforms for data collection. Other software such as epi-survey and form hub are also viable options for data management in resource-limited settings. With improvements in quality of Android phones, mobile and data service provision, and technical skill, we foresee health development practitioners and researchers in resource-limited settings relying more on paperless data management.

### Conclusions

Paperless data management has obvious benefits over paper-based data management and is found to be quite useful even in resource-limited settings with prospects for use in large-scale regional and national surveys, active and passive surveillance, and other epidemiology activities. As cellular networks improve, we are likely to experience an increasing shift from paper-based to paperless health management information systems.

## Abbreviations

CIRCLE: Climate Impact Research, Capacity and Leadership Enhancement
DfID: Department for International Development
GPS: global positioning system
GSM: global system for mobile communication
LGAs: Local Government Areas
ODK: Open Data Kit
SD: standard deviation
SIM: subscriber identity module
WHO: World Health Organization
